# Prediction of line heating deformation on sheet metal based on an ISSA–ELM model

**DOI:** 10.1038/s41598-023-28538-8

**Published:** 2023-01-23

**Authors:** Lei Li, Shukang Qi, Honggen Zhou, Lei Wang

**Affiliations:** School of Mechanical Engineering, Jiangsu University of Science and Technology, Zhenjiang, 212013 China

**Keywords:** Mechanical engineering, Statistical physics, thermodynamics and nonlinear dynamics

## Abstract

A prediction method based on an improved salp swarm algorithm (ISSA) and extreme learning machine (ELM) was proposed to improve line heating and forming. First, a three-dimensional transient numerical simulation of line heating and forming was carried out by applying a finite element simulation, and the influence of machining parameters on deformation was studied. Second, a prediction model for the ELM network was established based on simulation data, and the deformation of hull plate was predicted by the training network. Additionally, swarm intelligence optimization, particle swarm optimization (PSO), the seagull optimization algorithm (SOA), and the salp swarm algorithm (SSA) were studied while considering the shortcomings of the ELM, and the ISSA was proposed. Input weights and hidden layer biases of the ELM model were optimized to increase the stability of prediction results from the PSO, SOA, SSA and ISSA approaches. Finally, it was shown that the prediction effect of the ISSA–ELM model was superior by comparing and analyzing the prediction effect of each prediction model for line heating and forming.

## Introduction

The hull plate is an important part of a ship's main structure. Its function is to ensure a watertight hull and enable the ship to float in the water and provide transport function. There are two main manufacturing methods used for curved ship plates: cold forming and hot forming. In cold forming, loads are usually applied by hydraulic pressure or roller pressure to achieve the desired deformation state. Hot forming applies heat to a sheet metal surface, resulting in a temperature gradient, that eventually leads to local deformation of the plate. At present, the main method of machining nondevelopable surfaces is line heating and forming. Line-heating technology is currently used to make large hulls and complex curved surfaces. It has the advantages of a wide application range, low cost, and high flexibility and adaptability. At present, however, the technique is mostly done by hand by skilled workers. The process suffers from a lack of experienced workers, difficulty in training workers, heavy workloads, poor working environments and high labor intensity. Therefore it is necessary to study the relationship between processing parameters and sheet deformation.

The factors that can affect the deformation of a ship plate include the moving speed of the heat source, the heating intensity and the heating mode. Choi^1,2^ studied the temperature distribution of heated plates. The influence of machining parameters on temperature was also studied. The results showed that the peak temperature decreases with increasing heat source velocity. Qi^3^ believed that the heating path would produce uneven deformation. The phenomenon was studied by numerical simulation. Variable-speed line heating technology was also proposed. Zhu^4^ conducted a numerical study on the power distribution considering the coupling method of the temperature characteristics of materials. Huang^5^ proposed a dynamic mesh refinement method based on multilevel refinement, applied it to craft a transient thermodynamic solution for line heating, and proved the feasibility of the method. Xu^6^ derived a formula for the heat absorption efficiency of a plate based on the finite volume method. This provided a reference for analyzing the heat dissipation of a heat source in air. Akiyama^7^ studied the influence of interference between multiple heating wires on sheet deformation. Deng^8,9^ proposed an elastic finite element method for predicting the structural deformation of large-scale welding. The welding deformation of a curved plate structure was predicted by the elastic finite element method. Based on natural strain theory, Huang^10^ used a local solid model and a whole shell model to rapidly predict the deformation of laser welded thin plates. Han^11^ analyzed the deformation distribution of a heated zone and unheated zone based on single and multiple torches. Based on the above research, this paper carries out a line heating simulation experiment for sheet metal.

It takes considerable time to calculate complex thermoelastoplasticity by numerical simulation, as it requires higher computer performance. Combining line heating technology with an algorithm for generating machining parameters can greatly improve the process of predicting sheet metal forming. Shin^12^ established a BP neural network model for plates. The thickness of the plate and the heating speed were the input reference data, and the longitudinal displacement was the output reference data. This was used to predict the local deformation of line heating. Jin^13^ used experiments and simulations to establish a prediction equation of angle deformation according to the reference data of heat source strength, bending stiffness and dimension. Nguyen^14^ developed an artificial neural network model to predict heating parameters by setting an input and output training network. Based on a thermal analysis finite element model of the thermal forming process of ship outer plates, Zhang^15^ proposed applying a support vector machine (SVM) to predict local deformation under line heating. Shanbehzadeh^16^ conducted a study on early BC prevention based on an ML prediction system and the results showed that machine learning has good predictive power. Nopour^17^ chose machine learning techniques to conduct research into the prediction of COVID-19, a model that could help doctors achieve early detection and effective intervention and potentially reduce patient deaths. Therefore, machine learning has been used to build mathematical models that can reproduce real machining provide results similar to those in real work, be used to analyze results and can predict and optimize certain model problems. Huang^18,19^ proposed a simple and effective single-hidden-layer feedforward neural network learning algorithm. The extreme learning machine (ELM) algorithm^20^ offers a faster learning speed and better generalization performance. Its convergence speed is much faster than that of traditional methods, and it has good performance and has been applied in many fields^21^. The salp algorithm^22^ has optimized many problems in machine learning, engineering design and other fields in recent years. Yildiz^23^ analyzed the effectiveness and capability of the salp algorithm from the aspects of convergence speed, solution quality and robustness. If plate deformation prediction can be realized, the machining difficulty can be greatly reduced and the efficiency can be increased.

This paper introduces a line heating and forming simulation model. The properties of the experimental materials are defined. The relevant data were obtained through experiments. The ELM prediction model based on the improved salp swarm algorithm (ISSA) was established by studying the relevant algorithm theory. By comparing the evaluation indices, the five prediction models in this paper are compared and evaluated, and it is concluded that ISSA–ELM has a better effect.

## Line heating deformation

**Figure 1:**
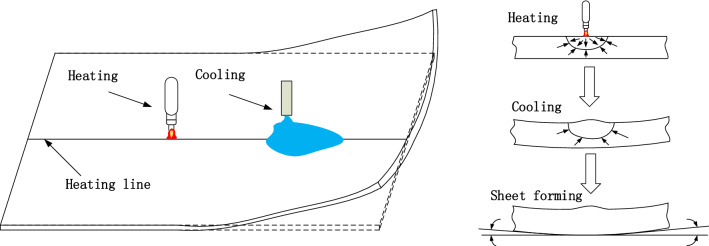
Principle of line heating and forming.

Line heating and forming technology refers to the use of combustible gases, electromagnetics, laser and other substances for line heating a steel plate. The heated area is then quickly cooled using water or air. The bending deformation of the steel plate is realized by the shrinkage stress of one part of the steel plate. Its forming principle is shown in Fig. 1. The forming results are affected by the temperature of the heat source, geometric reference data of the outer plate and processing reference data.

### Experimental principles

Line heating and forming is a method of shaping metal plates into complex three-dimensional shapes. This method is efficient and inexpensive. The gradient change in temperature is the main reason for the different bending degrees of sheet metal. In general, the difference between the mechanical and physical properties of metals is determined by temperature. As the temperature of the line heating zone increases, the standard bending strength, elasticity and thermal conductivity of steel decrease. However, the specific heat coefficient and expansion coefficient increase, particularly yield strength and Young’s modulus. The temperature drops rapidly above the critical temperature.

In the line heating process, the heat generated during sheet plastic deformation can be ignored. First, thermal analysis is performed. It is used create a temperature field and then used as a load for structural analysis.

The temperature field model for line heating and forming can be divided into two parts: the mathematical model of the heat flux of the gas source and the mathematical model of heat conduction. In the line heating and forming process, the gas heats the steel plate with a jet flame. The heat value on the unit cross-sectional area of the ship plate per unit time is also the data for the heat flux of the steel plate $q^{\prime \prime }$.

1 $$ q^{\prime \prime } = Q_{ch} A\eta { } $$

In the formula, $Q_{ch}$ is the flow of gas from the steel gun; $A$ is the combustion value of gas; and $\eta$ is the thermal efficiency.

In this study, the Gaussian heat source was chosen as the line heating heat source model due to its close proximity to the heat flow density distribution curve.

When heating a steel plate, the heat distribution is different at each location. The amount of heat absorbed per unit time at a given location is $q$:

2 $$ q = 0.95 \times {\raise0.7ex\hbox{${q^{\prime \prime } }$} \!\mathord{\left/ {\vphantom {{q^{\prime \prime } } {r_{0}^{2} }}}\right.\kern-0pt} \!\lower0.7ex\hbox{${r_{0}^{2} }$}} \times {\text{ep}}\left[ { - 3\left( {{\raise0.7ex\hbox{$r$} \!\mathord{\left/ {\vphantom {r {r_{0} }}}\right.\kern-0pt} \!\lower0.7ex\hbox{${r_{0} }$}}} \right)^{2} } \right] $$

where $r_{0}$ is the flame heating radius and $r$ is the distance between the point to be heated and the flame heating center.

The line heating process can be regarded as three-dimensional nonlinear transient analysis, and its heat conduction mathematical model is:

3 $$ \rho c\frac{\partial T}{{\partial t}} = \frac{\partial }{\partial x}\left( {\lambda \frac{\partial T}{{\partial x}}} \right) + \frac{\partial }{\partial y}\left( {\lambda \frac{\partial T}{{\partial y}}} \right) + \frac{\partial }{\partial z}\left( {\lambda \frac{\partial T}{{\partial z}}} \right) $$

In the formula, $\rho$ is the density of the steel; $c$ is the specific heat capacity of the steel; $\lambda$ is the thermal conductivity of the steel; $T$ is the temperature of the steel; and $t$ is the heating time.

**Figure 2:**
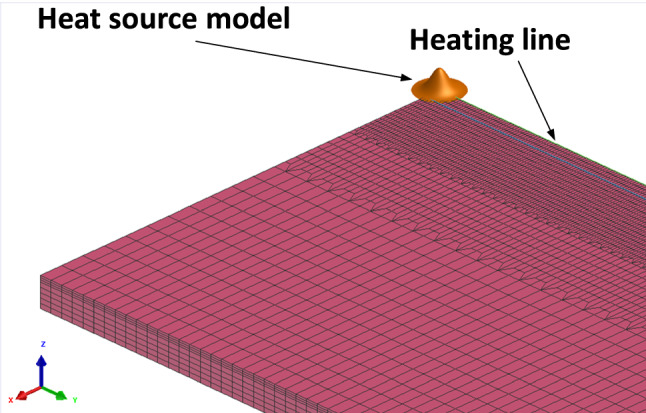
Heat source model.

Figure 2 shows the heat source model established in this study. Both the free convection and radiation boundary conditions are satisfied. In mechanical analysis, these boundary conditions are limited to zero displacement at the centerline of the metal plate to avoid rigid body motion.

**Figure 3:**
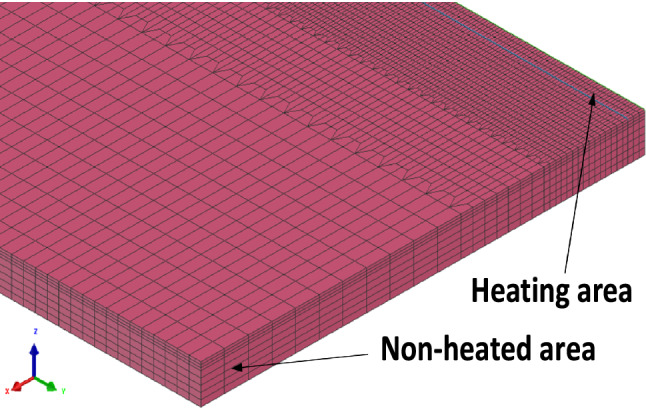
Plate finite element model.

The process of linear heating is a thermodynamic coupling phenomenon. In this study, a three-dimensional finite element model was established to simulate the shaping process of sheet metal. The simulation software SYSWELD was used to simulate the shaping process. The finite element model of the steel plate is shown in Fig. 3 for grid division. In the numerical model, the position close to the heat source is used as a fine grid, while the position far from the heat source is used as a coarse grid. The aim is to obtain accurate temperature and stress gradients and reduce calculation time.

### Experimental parameter setting

The dimensions of the steel plate chosen for this experiment were 300mm × 400 mm × hmm ($h \in \left[ {10,14} \right]$). The heating line was located in the center line of the plate width direction, and the heat source speed was set at 6–15 mm/s.

Q345 is widely used in ships, vehicles and construction because of its moderate carbon content, superior overall properties, good strength, plasticity and reliability. In this paper, Q345 steel is selected for study and its properties vary with temperature as shown in Table 1.

**Table 1:** Q345 steel performance parameters.

Temperature/℃	Thermal conductivity/[W/(mK)]]	Density/[kg/m^3^]	Specific heat capacity/[J/(kg × K)]	Poisson’s ratio	Expansion factor/(°C^−1^)	Modulus of elasticity/MPa	Young’s modulus/MPa
20	50	7820	460	0.28	1.1 × 10^−5^	2.05 × 10^5^	2.2 × 10^2^
250	47	7700	480	0.29	1.2 × 10^−5^	1.87 × 10^5^	1.75 × 10^2^
500	40	7610	530	0.31	1.39 × 10^−5^	1.5 × 10^5^	0.8 × 10^2^
750	27	7550	675	0.35	1.48 × 10^−5^	0.7 × 10^5^	0.4 × 10^2^
1000	30	7490	670	0.4	1.34 × 10^−5^	2 × 10^−3^	0.1 × 10^2^
1500	45	7350	660	0.49	1.33 × 10^−5^	1.5 × 10^−3^	0.1 × 10^−10^

### Experimental data

**Figure 4:**
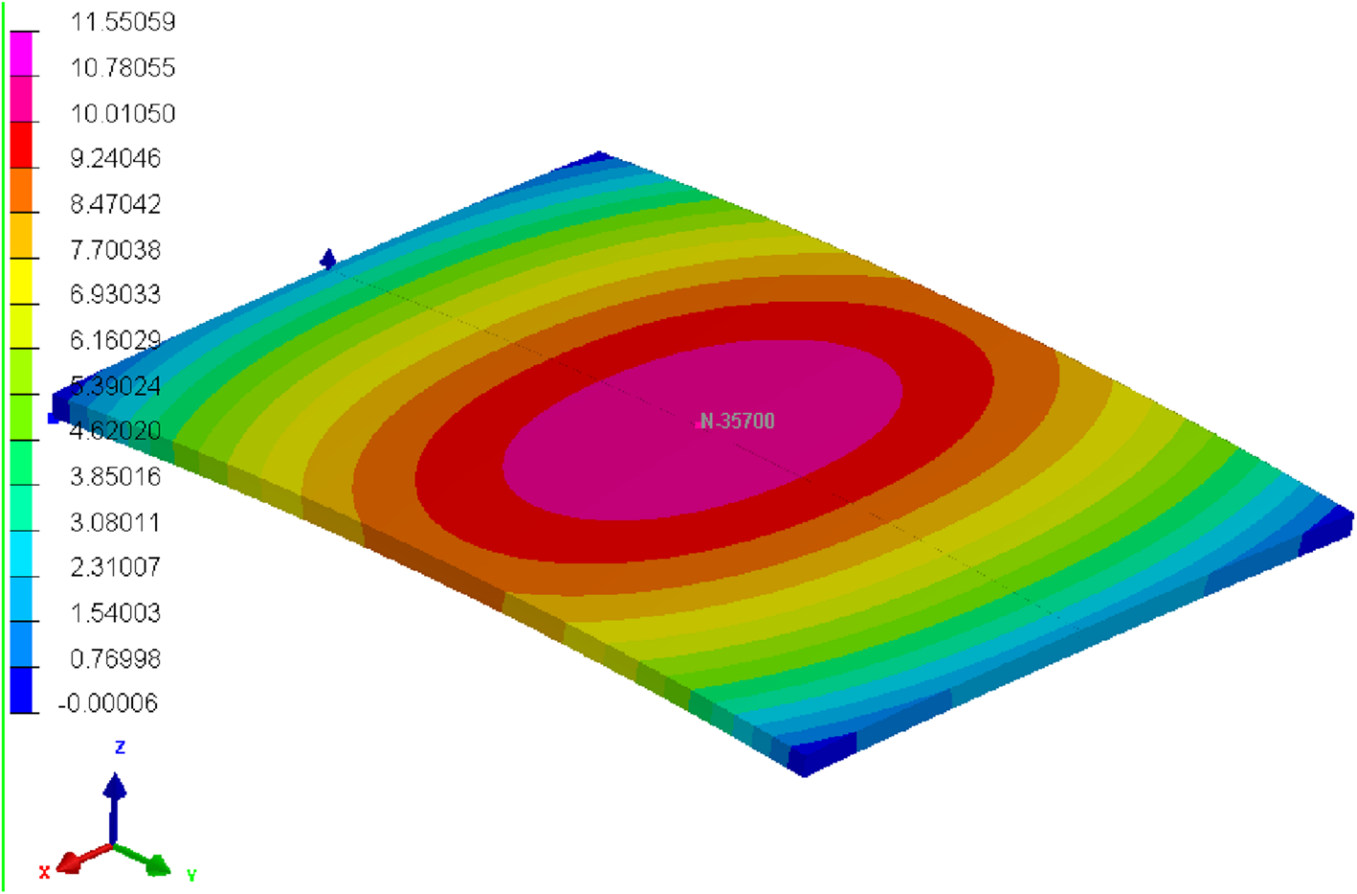
Curved forming shape.

There are 90 groups of experiments in this paper. The first column shows the experiment number. The next four columns represent the parameters of the feature. The last column represents the result. The experimental data are presented in Table 2. The formed shape is shown in Fig. 4.

**Table 2:** Experimental data.

Experiment number	Heat source speed (mm/s)	Energy input (J/mm)	Thickness (mm)	Number of heats	Amount of deformation (mm)
1	6	600	14	1	5.785
2	7	600	14	1	5.771
3	8	600	14	1	5.760
4	9	600	14	1	5.748
5	10	600	14	1	5.727
6	11	600	14	2	7.712
7	12	600	14	2	7.696
…	…	…	…	…	…
87	12	1000	10	2	13.506
88	13	1000	10	2	13.443
89	14	1000	10	2	13.383
90	15	1000	10	1	11.322

## Establishment of the machine learning model

The deformation degree of line heating is affected by many factors such as material and processing parameters. The machine learning method is used to explore the rules between different input variables and output results to achieve deformation prediction. An extreme learning machine is a new kind of special single hidden layer feed forward neural network. Its network structure is simple. It can randomly generate input weights and hidden layer biases. The network training time is very fast. However, its stability and generalization ability are slightly poor. The input weights and the bias of the hidden layer in an ELM are randomly selected. Randomly generated values may not be good for learning samples, which makes the learning effect unstable. To obtain a stable prediction effect, the swarm intelligence optimization algorithm can be used to optimize the input weight and the bias of the hidden layer of an ELM. This paper presents an optimized ELM based on an improved slap swarm algorithm, to find the best solution. Therefore, the prediction error and computational efficiency of extreme learning machines based on the SOA, PSO, SSA and ISSA are compared. Choosing a better optimization algorithm among these methods allows the ELM to calculate more efficiently to achieve accurate prediction.

### Extreme learning machine

**Figure 5:**
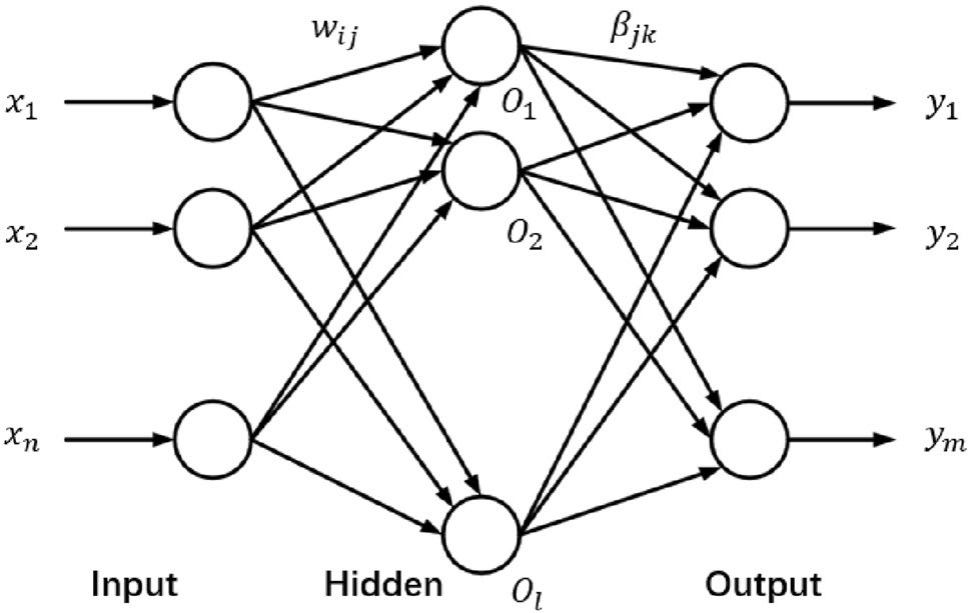
ELM network structure.

Figure 5 shows the structure of a typical single hidden layer feedforward neural network. It is composed of an input layer, hidden layer and output layer. Among them, $n$ neurons in the input layer correspond to $N$ input variables. The hidden layer has $1$ neuron. The output layer has $m$ neurons corresponding to $m$ output variables. To maintain generality, let the connection weight between the input layer and the hidden layer be $w$:

4 $$ w = \left[ {\begin{array}{*{20}c} {\begin{array}{*{20}c} {w_{11} } & {w_{12} } \\ {w_{21} } & {w_{22} } \\ \end{array} } & \cdots & {\begin{array}{*{20}c} {w_{1n} } \\ {w_{2n} } \\ \end{array} } \\ \vdots & \ddots & \vdots \\ {\begin{array}{*{20}c} {w_{l1} } & {w_{l2} } \\ \end{array} } & \cdots & {w_{ln} } \\ \end{array} } \right] $$

where $w_{n}$ denotes the weight of the connection between the $i$th neuron in the input layer and the $j$th neuron in the hidden layer.

Assume that the connection weight between the implied layer and the output layer is $\beta$:

5 $$ \beta = \left[ {\begin{array}{*{20}c} {\begin{array}{*{20}c} {\beta_{11} } & {\beta_{12} } \\ {\beta_{21} } & {\beta_{22} } \\ \end{array} } & \cdots & {\begin{array}{*{20}c} {\beta_{1m} } \\ {\beta_{2m} } \\ \end{array} } \\ \vdots & \ddots & \vdots \\ {\begin{array}{*{20}c} {\beta_{l1} } & {\beta_{l2} } \\ \end{array} } & \cdots & {\beta_{lm} } \\ \end{array} } \right] $$

where $\beta_{jk}$ denotes the connection weight between the $j$th neuron of the hidden layer and the $k$th neuron of the output layer.

Assume that the threshold $b$ of the implicit layer neuron is:

6 $$ b = \left[ {\begin{array}{*{20}c} {\begin{array}{*{20}c} {b_{1} } \\ {b_{2} } \\ \end{array} } \\ \cdots \\ {b_{l} } \\ \end{array} } \right] $$

Assume that the input matrix X and output matrix Y of the training set with Q samples are:

7 $$ X = \left[ {\begin{array}{*{20}c} {\begin{array}{*{20}c} {x_{11} } & {x_{12} } \\ {x_{21} } & {x_{22} } \\ \end{array} } & \cdots & {\begin{array}{*{20}c} {x_{1Q} } \\ {x_{2Q} } \\ \end{array} } \\ \vdots & \ddots & \vdots \\ {\begin{array}{*{20}c} {x_{n1} } & {x_{n2} } \\ \end{array} } & \cdots & {x_{nQ} } \\ \end{array} } \right] $$

8 $$ Y = \left[ {\begin{array}{*{20}c} {\begin{array}{*{20}c} {y_{11} } & {y_{12} } \\ {y_{21} } & {y_{22} } \\ \end{array} } & \cdots & {\begin{array}{*{20}c} {y_{1Q} } \\ {y_{2Q} } \\ \end{array} } \\ \vdots & \ddots & \vdots \\ {\begin{array}{*{20}c} {y_{m1} } & {y_{m2} } \\ \end{array} } & \cdots & {y_{mQ} } \\ \end{array} } \right] $$

Assume that the activation function of the neuron in the hidden layer is g(x), then from the network structure of the ELM, the output T of the network is:

$$ T = \left[ {t_{1} , \cdots ,t_{Q} } \right]_{m \times Q} , $$

9 $$ t_{j} = \left[ {t_{1j} , \ldots ,t_{mj} } \right]^{T} = \left[ {\begin{array}{*{20}c} {\begin{array}{*{20}c} {\mathop \sum \limits_{i = 1}^{t} \beta_{i1} g\left( {w_{i} x_{i} + b_{i} } \right)} \\ {\mathop \sum \limits_{i = 1}^{t} \beta_{i2} g\left( {w_{i} x_{i} + b_{i} } \right)} \\ \end{array} } \\ \cdots \\ {\mathop \sum \limits_{i = 1}^{t} \beta_{im} g\left( {w_{i} x_{i} + b_{i} } \right)} \\ \end{array} } \right]_{m \times 1} \;\left( {j = 1,2, \ldots ,Q} \right) $$

The above equation can be expressed as: $H\beta = T^{\prime }$.

where $T^{\prime }$ is the transpose matrix of $T$ and $H$ is the output matrix of the hidden layer of the neural network in the following form.

10 $$ \left( {w_{1} , \ldots ,w_{i} ,b_{1} , \ldots ,b_{l} ,x_{1} , \ldots ,x_{Q} } \right) = \left[ {\begin{array}{*{20}c} {\begin{array}{*{20}c} {g\left( {w_{1} {*}x_{1} + b_{1} } \right)} & {g\left( {w_{2} {*}x_{1} + b_{2} } \right)} \\ {g\left( {w_{1} {*}x_{2} + b_{1} } \right)} & {g\left( {w_{2} {*}x_{2} + b_{2} } \right)} \\ \end{array} } & {\begin{array}{*{20}c} \cdots & {g\left( {w_{l} {*}x_{1} + b_{l} } \right)} \\ \cdots & {g\left( {w_{l} {*}x_{2} + b_{l} } \right)} \\ \end{array} } \\ {\begin{array}{*{20}c} \vdots & \vdots \\ {g\left( {w_{1} {*}x_{Q} + b_{1} } \right)} & {g\left( {w_{2} {*}x_{Q} + b_{2} } \right)} \\ \end{array} } & {\begin{array}{*{20}c} \ddots & \vdots \\ \cdots & {g\left( {w_{l} {*}x_{Q} + b_{l} } \right)} \\ \end{array} } \\ \end{array} } \right]_{{Q{*}l}} $$

### Salp swarm algorithm

#### Population initialization

A chain of $n$ salps is considered as a population. The individual position of each salp is represented as a $D$ dimensional vector. The location of salps can be expressed as $X = \left[ {X_{n1} ,X_{n2} , \ldots ,X_{nD} } \right]^{T} ,n = 1,2, \ldots ,N$. The function that requires the maximum value is called the fitness function. An individual can be substituted as the independent variable in the formula, for the position of the salp swarm to calculate its corresponding. The position of this individual is called the food position and is denoted as $F = \left[ {F_{1} ,F_{2} , \ldots ,F_{D} } \right]^{T}$, the upper limit of the search space is $ub = \left[ {ub_{1} ,ub_{2} , \ldots ,ub_{D} } \right]^{T}$,the lower limit of the search space is $lb = \left[ {lb_{1} ,lb_{2} , \ldots ,lb_{D} } \right]^{T}$, and the population of the SSA. The random initialization formula is:

11 $$ X_{{N{*}D}} = R\left( {N,D} \right){*}\left( {ub - lb} \right) + lb $$

where $X_{N*D}$ denotes the salp swarm position vector and $R\left( {N,D} \right)$ denotes the $N*D$ dimensional random vector.

#### Update leader positions

The leader salp swarm is the first vector in the X-matrix. In the standard SSA, the leader leads the movement of the whole salp colony. Its next position will be in the direction of food in some way. The leader update strategy is calculated by Eq. (12).

12 $$ X_{d}^{1} = \left\{ {\begin{array}{*{20}c} {F_{d} + c_{1} \left[ {\left( {ub_{d} - lb_{d} } \right)c_{2} + lb_{d} } \right],c_{3} > 0.5} \\ {F_{d} - c_{1} \left[ {\left( {ub_{d} - lb_{d} } \right)c_{2} + lb_{d} } \right],c_{3} \le 0.5} \\ \end{array} } \right. $$

where $X_{d}^{1}$ is the leader position. $ub_{d} ,lb_{d}$ denote the upper and lower search limits of the individual leader in dimension $d$. $c_{1} ,c_{2}$ denote random numbers taking the values [0,1]. $c_{1}$ is used to control the search ability and exploitation ability of the whole group, and $c_{2}$ determines the length of the move.$c_{3}$ is the search balancing factor, which determines the positive and negative directions of movement and is used to balance global and local search capabilities. Ultimately, this increases the randomness and diversity of leaders.

#### Update follower positions

A follower of the salp is a salp that follows the movement of the leader. The position of the $m$th follower in the next iteration is determined by its own position in the current iteration and the position of the previous salp. The initial position, velocity, and acceleration directly affect the position of the follower. The updated positions can be obtained according to Newton's equations of motion, as shown in Eqs. (13, 14 and 15):

13 $$ X_{d}^{m} = 0.5at_{a}^{2} + v_{0} t_{a} + X_{d}^{m} ,\left( {i \ge 2} \right) $$

14 $$ R = \frac{1}{2}\left( {X_{d}^{m - 1} - X_{d}^{m} } \right) $$

15 $$ X_{d}^{{m^{\prime } }} = X_{d}^{m} + R = \frac{1}{2}\left( {X_{d}^{m} + X_{d}^{m - 1} } \right) $$

where $a$ means acceleration; $v_{0}$ means the initial velocity; $t_{a}$ means the iteration step length; $R$ means the distance of movement; and $X_{d}^{{m^{\prime } }} ,X_{d}^{m}$ denotes the $m$ th follower's $d$ th dimensional position after and before the update.

### Improved salp swarm algorithm

#### Attenuation factor

In this paper, we introduce an attenuation factor into the SSA. Therefore, the leader position update range gradually decreases with the increase in the number of iterations, avoiding falling into local extremes in the early stage of convergence. Then, it gets closer to the optimal value in the late stage of accuracy to achieve higher solution accuracy. The leadership of the position update formula with the addition of the decay factor is shown in Eq. (16).

16 $$ X_{d}^{1} = \left\{ {\begin{array}{*{20}c} {A\left( l \right)[F_{d} + c_{1} \left( {\left( {ub_{d} - lb_{d} } \right)c_{2} + lb_{d} } \right)],c_{3} > 0.5} \\ {A\left( l \right)[F_{d} - c_{1} \left( {\left( {ub_{d} - lb_{d} } \right)c_{2} + lb_{d} } \right)],c_{3} \le 0.5} \\ \end{array} } \right. $$

where the decay factor A(l), which controls the search range, is a nonlinear decreasing function defined as in Eq. (17):

17 $$ A\left( l \right) = e^{{ - \left( {2l/T} \right)^{2} }} $$

where $l$ is the number of current iterations. $T$ denotes the maximum number of iterations.

In the early stage of convergence, the search range is not restricted. Individuals can fully move around the globe to give full play to the global search capability of the algorithm and avoid falling into local extremes. To achieve higher accuracy, in the late stages of convergence, the search range gradually decreases as the individual gets closer to the optimal value using individual precision search within a restricted range to enhance local search capabilities.

#### Adaptive inertia weights

To enhance local search, the algorithm searches for larger weights in the early stages that can enhance the global search and smaller adaptive weights in the late stages of the search that can introduce adaptive weights, as shown in Eq. (18):

18 $$ \omega = \lambda^{{ - \varphi {*}l/T}} {\text{*cos}}\left( {\pi l} \right) $$

By introducing the above equation into the old follower update equation, the new follower update equation is Eq. (19):

19 $$ X_{d}^{{m^{\prime}}} = \frac{1}{2}\left( {X_{d}^{m} + \omega X_{d}^{m - 1} } \right) $$

when $\lambda = 2$ and $\varphi = 4/3$, the value of $\omega$ gradually converges. This can improve the computational complexity. The search range and the population diversity are increased in the early stage of the algorithm.

The improved implementation of the Salp Swarm Algorithm flows as follows:

- *Step 1* Set the population size $N$, the number of iterations Iteration, the dimension $D$, and the upper and lower boundaries.
- *Step 2* Initialize the position of individuals in the salp swarm group. Then calculate the fitness value fitness of each individual to determine the position of the individual with the smallest fitness value as the food position.
- *Step 3* Generate decay factor $A\left( l \right)$ and update the leadership of the position.
- *Step 4* Add adaptive inertia weights to update the follower positions.
- *Step 5* If it is smaller than the current food position, calculate the updated individual adaptation value and update the food position.
- *Step 6* Determine whether the current iteration count reaches the preset iteration count. If it is reached, end the iteration; otherwise, return to Step3.
- *Step 7* Output the Food Position and the fitness value on that position.

### The ELM optimized by ISSA

**Figure 6:**
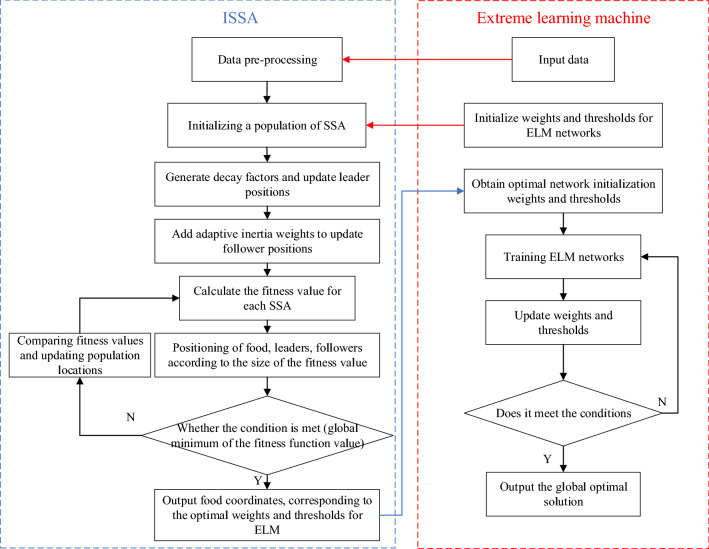
Flowchart of the optimized ELM based on the ISSA.

Through the learning of the SSA we found that due to its unconstrained search range of position updates and the small influence weight of elite individuals, the SSA cannot perform an accurate search in the late iteration, and the followers cannot assist well in individual position updates. In this paper, we propose an improved SSA to optimize the regression prediction of line heating and forming for extreme learning machines. First, an attenuation factor is added to enhance local exploitation later in the iteration. Then, adaptive inertia weights are added to enhance the global search capability in the early stages and the local search capability in the later stages. The flow of the improved SSA for optimizing the extreme learning machine is shown in Fig. 6.

### Model accuracy evaluation

To further evaluate the prediction performance of the model, the following three indicators are chosen to assess the accuracy of the model: the fitness value $E$, the mean squared error $MSE$ and the coefficient of determination $R^{2}$, whose equations are shown in Eqs. (20, 21 and 22) respectively.

20 $$ E = \sqrt {\frac{{\mathop \sum \nolimits_{d = 1}^{D} \left( {y_{d} \left( n \right) - y_{d}^{*} \left( n \right)} \right)^{2} }}{D}} { } $$

21 $$ MSE = \frac{1}{n}\mathop \sum \limits_{n = 1}^{D} \left( {y_{d} \left( n \right) - y_{d}^{*} \left( n \right)} \right)^{2} $$

22 $$ R^{2} = \frac{{\left( {n\mathop \sum \nolimits_{n = 1}^{D} y_{d}^{*} \left( n \right){*}y_{d} \left( n \right) - \mathop \sum \nolimits_{n = 1}^{D} y_{d}^{*} \left( n \right){*}\mathop \sum \nolimits_{n = 1}^{D} y_{d} \left( n \right)} \right)^{2} }}{{\left( {n\mathop \sum \nolimits_{n = 1}^{D} (y_{d}^{*} \left( n \right))^{2} - \left( {\mathop \sum \nolimits_{n = 1}^{D} y_{d}^{*} \left( n \right)} \right)^{2} } \right)\left( {n\mathop \sum \nolimits_{n = 1}^{D} \left( {y_{d} \left( n \right)} \right)^{2} - \left( {\mathop \sum \nolimits_{n = 1}^{D} y_{d} \left( n \right)} \right)^{2} } \right)}} $$

where $y_{d}^{*} \left( n \right)$ denotes the network training output, $y_{d} \left( n \right)$ denotes the desired output, and $D$ is the training data length.

## Results and discussion

In this paper, prediction models based on an ELM, PSO-ELM, SOA-ELM, SSA-ELM and ISSA–ELM are developed. The prediction performance of each model is shown in the figure.

**Figure 7:**
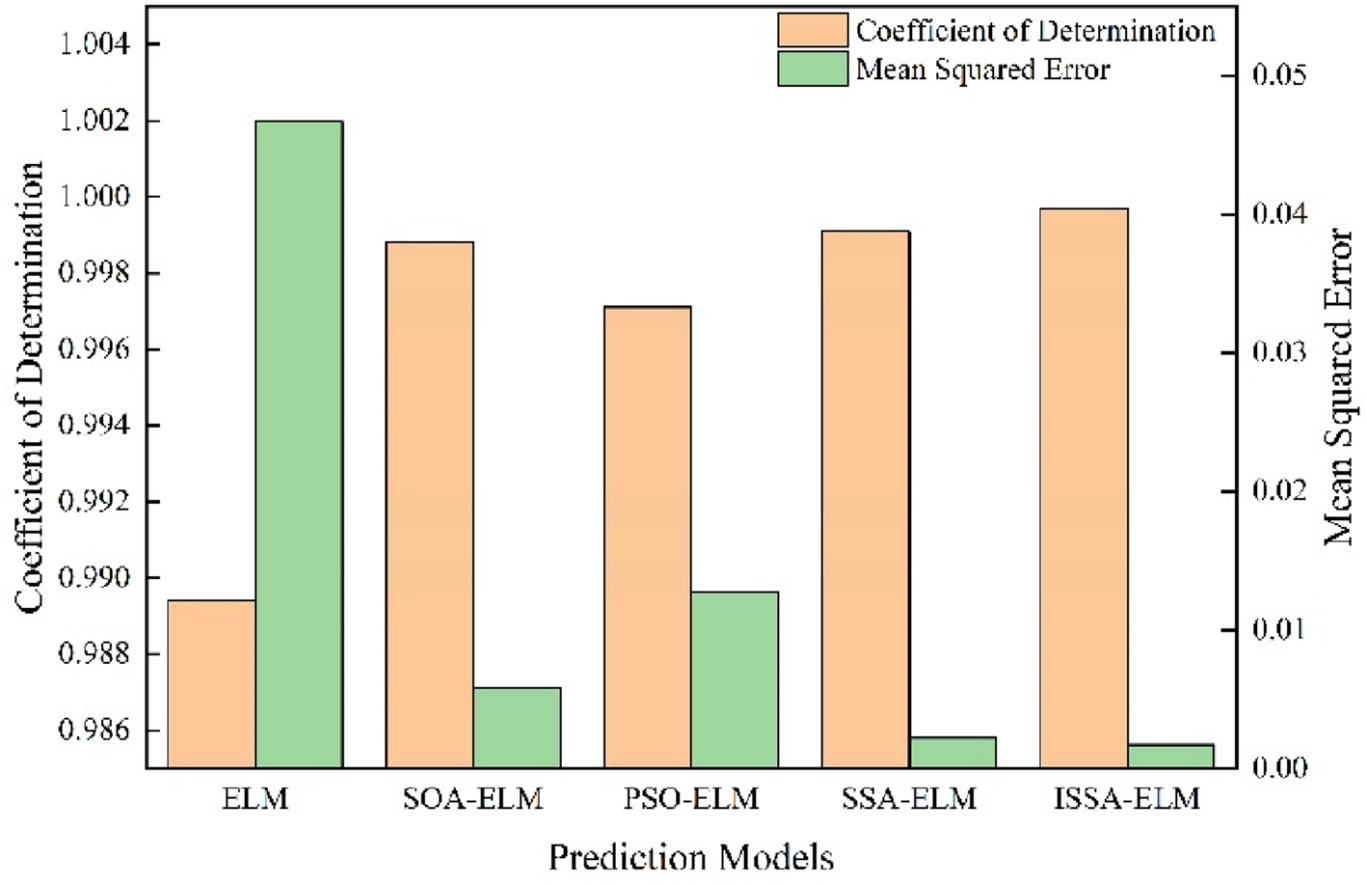
Comparing the mean squared errors and coefficients of determination of the different models.

The closer the coefficient of determination is to 1, the better the prediction performance of the model. Figure 7 shows the mean square error and coefficient of determination for all prediction models used to predict line heating deformation. A comparative analysis shows that the initial ELM algorithm model has a relatively low coefficient of determination. The models optimized by the optimization algorithm all have coefficients of determination close to 1. It is obvious that ISSA–ELM has a better coefficient of determination.

The closer the mean square error is to 0, the better the performance of the model. Figure 7 shows that the mean square error of the original ELM model is higher than that of the other four models, with a mean square error of 4.67E−2. The optimized ELM models all perform well with mean square errors of 5.8E−3, 1.27E−2, 2.2E−3 and 1.7E−3.

**Figure 8:**
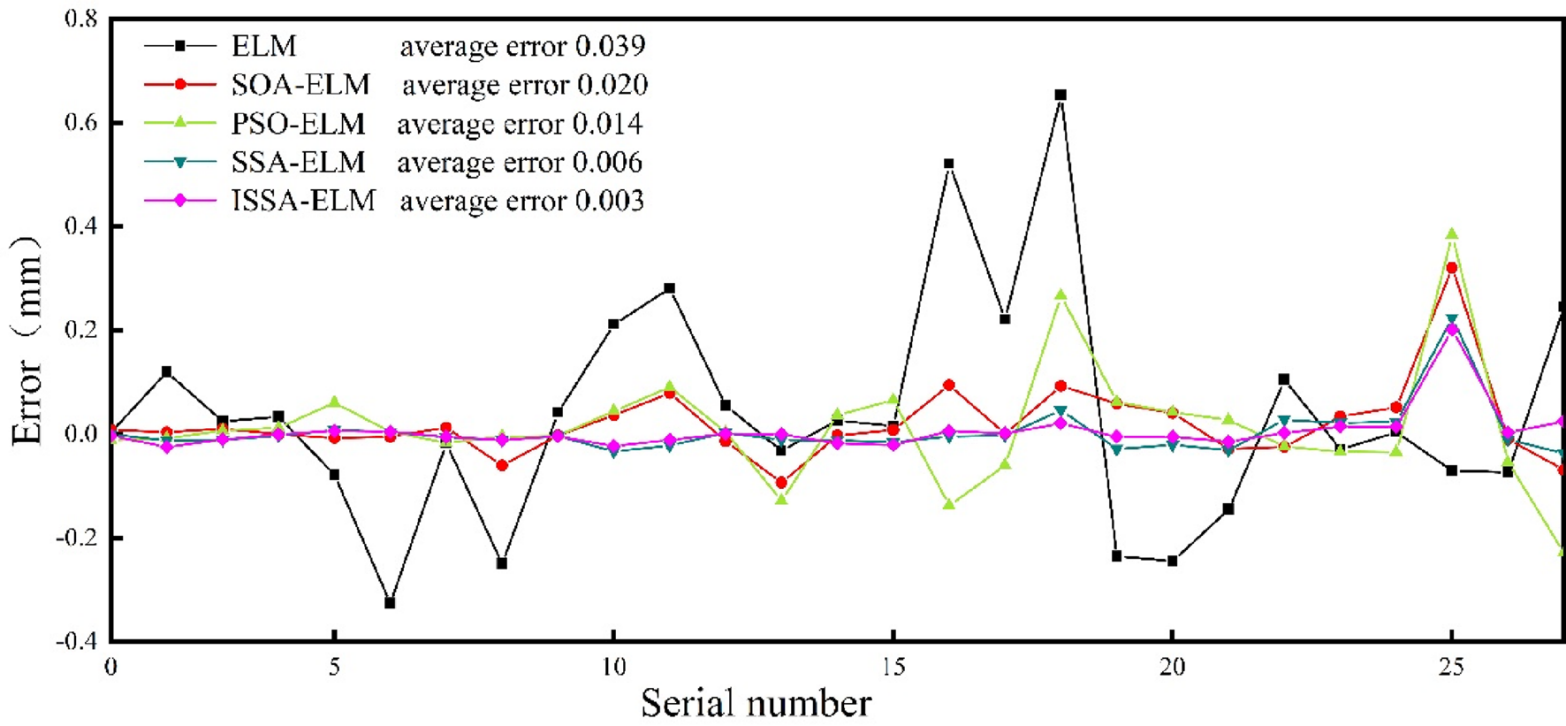
Comparing the prediction errors of different models.

The experimental values of the errors and mean errors between the predictions of the five prediction models in this paper are shown in Fig. 8. The average prediction error of the ELM is 0.039 mm, which is significantly higher than the average error of the other models. It is clear that the initial ELM algorithm did not find the global optimal solution. The mean error of the ELM algorithm was significantly reduced after optimization by the optimization algorithm. The ISSA–ELM model had the smallest mean prediction error of all the models, so its prediction performance was optimal.

**Figure 9:**
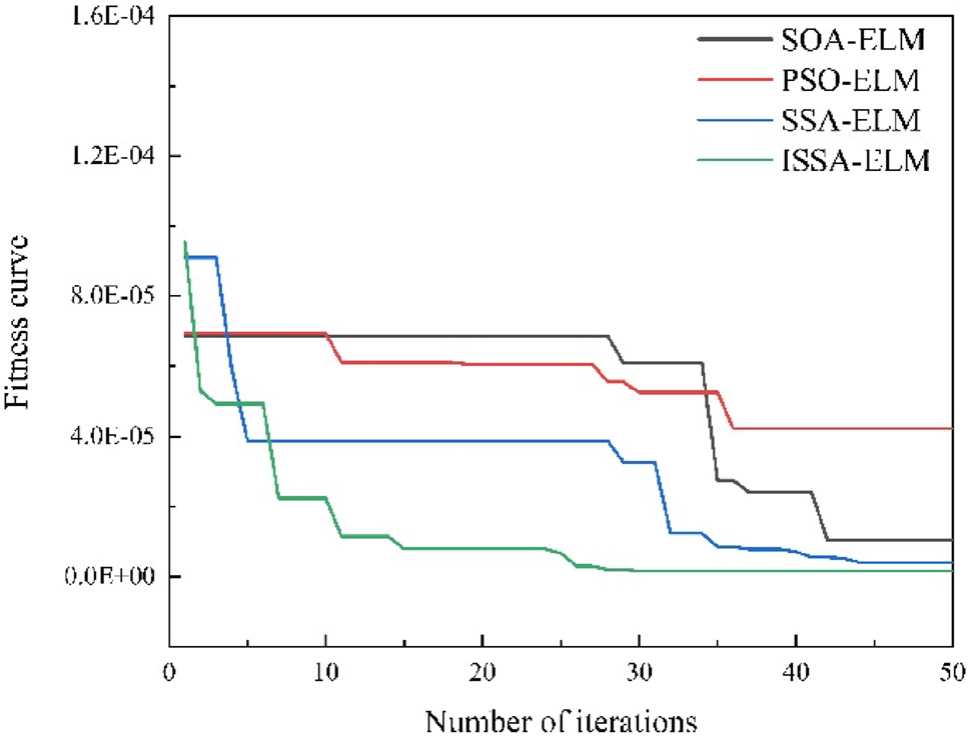
Comparing the fitness curves of different models.

The fitness curves for the prediction models used to predict line heating and forming deformation SOA-ELM, PSO-ELM, SSA-ELM and ISSA–ELM are shown in Fig. 9. The fitness function value is the MSE of the error in the training set: $fitness = argmin\left( {MSE_{pridect} } \right)$. In the SOA-ELM model, after 41 iterations, the systematic error is stabilized at approximately 1.0E−5. In the PSO-ELM model, after 35 iterations, the systematic error is stabilized at approximately 4.0E−5. In the SSA-ELM model, after 42 iterations, the system error is stabilized at approximately 5.0E−6. In the ISSA–ELM model, after 30 iterations, the systematic error is stabilized at approximately 2.0E−6. The results show that the ISSA–ELM model proposed in this paper has a faster optimization speed and higher convergence accuracy, and the improved sheath algorithm significantly improves the learning efficiency of the ELM model.

**Figure 10:**
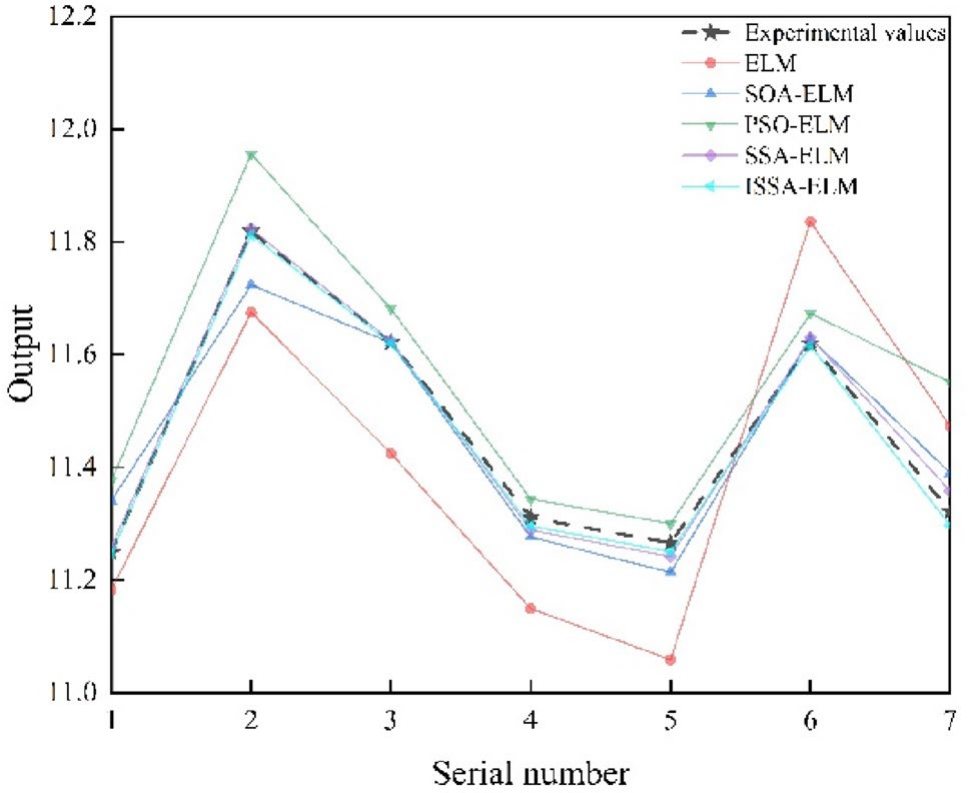
Predicted versus desired output for each model.

The comparison between the prediction output and experimental values of the five prediction models in this paper is shown in Fig. 10. The overall trend of each model is close to the experimental value. It is obvious in Fig. 10 that the prediction effect of the original ELM was significantly different from the experimental value. The smaller the relative error is, the better the prediction effect. The prediction effect of the original ELM was poor, and the average relative error of ELM was 1.4%. The average relative error of the ELM prediction model optimized by the optimization algorithm is much smaller than that of the original ELM. Their average relative errors are 0.4%, 0.8%, 1.4%, 0.1% and 0.08%. Among them, the predicted output of the ISSA–ELM model proposed in this paper is closer to the experimental value. This indicates that the model is superior.

This paper addresses the characteristics of the line heating forming process. Using finite element simulation tools. A finite element simulation model based on thermoelasticity was developed. On this basis, process parameters and forming data were obtained. A machine learning method was introduced to predict the deformation of the shipboard. According to the shortcomings of the ELM, an improved salp swarm optimization algorithm was proposed for improving the ELM. The results show that the ISSA–ELM-based line heating deformation prediction model proposed in this paper has certain guiding significance for processing production.

## Conclusions

An algorithm model based on an improved salp swarm algorithm optimized extreme learning machine is used to predict line heating and forming. This has certain guidance significance for the reasonable prediction of deformation. The main conclusions are drawn as follows.

1. Using an ELM as the basic framework, the optimized prediction models were obtained by the seagull algorithm, the particle swarm algorithm, the salp swarm algorithm and the improved salp swarm algorithm. The prediction model based on the ISSA–ELM is found to be more suitable for line heating and forming deformation prediction by comparison.
2. The SSA is optimized by introducing attenuation factors and adaptive inertia weights, and the results show that the prediction performance of ISSA–ELM is better.
3. The results obtained with a single curvature flat plate are only applicable to a small sample and a small number of inputs. In a subsequent study, more material properties will be added to increase the number of inputs, based on simulation experiments as samples, to study double curvature plate deformation prediction based on the proposed prediction model.

## Data Availability

The data that support the findings of this study are available from Jiangsu University of Science and Technology but restrictions apply to the availability of these data, which were used under license for the current study and are not publicly available. Data are however available from the authors upon reasonable request and with permission from Jiangsu University of Science and Technology.
